# Typical response of CD14^++^CD16^–^ monocyte to knee synovial derived mediators as a key target to overcome the onset and progression of osteoarthritis

**DOI:** 10.3389/fmed.2022.904721

**Published:** 2022-08-29

**Authors:** Nik Syazana Izyan Saffery, Krishnamurithy Genasan, Chee Ken Chan, Khairul Anwar Ayob, Seow Hui Teo, Mohamed Zubair Mohamed Al-Fayyadh, Iekhsan Othman, Syafiq Asnawi Zainal Abidin, Murali Malliga Raman, Hanumantha Rao Balaji Raghavendran, Tunku Kamarul

**Affiliations:** ^1^Department of Orthopedic Surgery, National Orthopedic Centre of Excellence for Research and Learning (NOCERAL), Faculty of Medicine, University of Malaya, Kuala Lumpur, Malaysia; ^2^Department of Physiology, Faculty of Medicine, University of Malaya, Kuala Lumpur, Malaysia; ^3^Mahkota Medical Centre, Jalan Merdeka, Melaka, Malaysia; ^4^Jeffrey Cheah School of Medicine and Health Sciences, Monash University Malaysia, Jalan Lagoon Selatan, Selangor, Malaysia; ^5^Biomaterials Laboratory, Faculty of Clinical Research, Central Research Facility, Sri Ramachandra Institute of Higher Education and Research, Chennai, India; ^6^Advanced Medical and Dental Institute, University Sains Malaysia, Penang, Malaysia

**Keywords:** osteoarthritis, synovium, inflammation, MALDI-imaging, proteomics, monocytes

## Abstract

**Objective:**

Synovitis with increased infiltration of immune cells is observed in osteoarthritis (OA). Given the inflammatory condition of synovitis, we explored the protein profile of OA synovium (OAS) and its effect on circulating monocytes activation, migration, and functional commitments.

**Methods:**

Knee-synovium was acquired from end-stage OA (*N* = 8) and trauma patients (Trauma baseline control: TBC; *N* = 8) for characterization using H&E histology, IHC (iNOS), LCMS-QTOF, and MALDI-imaging. Response of peripheral blood monocytes to OAS conditioned-media (OACM) was observed using transwell (*n* = 6). The migrated cells were captured in SEM, quantified using phase-contrast microphotographs, and their activation receptors (CCR2, CXCR2, CX3CR1, and CD11b), pro-inflammatory genes, and phagocytic potential were studied using flow cytometry, gene expression array/qPCR, and latex beads (LB) phagocytosis assay, respectively.

**Results:**

The Venn diagram displayed 119 typical proteins in OAS, while 55 proteins in TBCS. The STRING protein network analysis indicated distinctive links between proteins and gene ontology (GO) and revealed proteins associated with leukocyte-mediated immunity in OAS as compared to TBC. The MALDI-imaging showed typical localized proteins at 2234.97, 2522.61, 2627.21, 3329.50, and 3539.69 *m*/*z* and IHC confirmed pro-inflammatory iNOS expression in OA synovium. CD14^++^CD16^–^ classical monocytes significantly migrated in OACM and expressed CCR2, CXCR2, and CD11b receptors, TNFRSF11A, MAPK1, S100A8, HSPB1, ITGAL, NFATC1, IL13RA1, CD93, IL-1β, TNF-α, and MYD88 genes and increased LB uptake as compared to SFM.

**Conclusion:**

Our findings suggest that the differential protein profile of OA synovium and the classical monocytes migrated, activated, and functionally committed in response to these mediators could be of therapeutic advantage.

## Graphical Abstract

The peripheral blood CD14^ + +^CD16^–^ monocyte subtype migration in response to knee synovium derived mediators. 
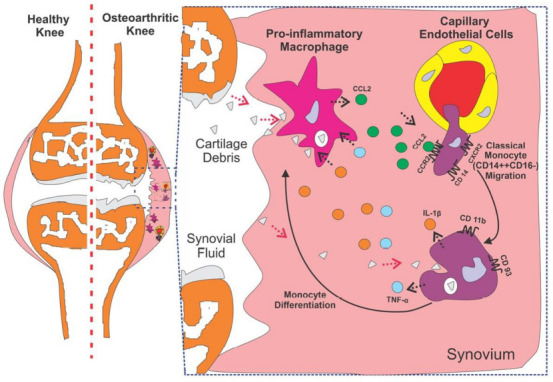

## Introduction

In osteoarthritis (OA) pathogenesis, the most accepted hypothesis is the activation of synoviocytes, primarily synovial macrophages, in response to internalized cartilage debris in synovium derived from worn-out cartilage (1). The activated macrophages prone to release pro-inflammatory mediators, including IL-1β, IL-6, and TNF, causing infiltration of immune cells from peripheral blood and subsequent acute alteration in the synovium (2). The recurrent inflammation due to the attempt to remove cartilage debris by macrophages contributes to an inflammatory vicious cycle. This leads to the development of low-grade inflammation in the synovium contributing to synovitis (3). Although the characteristic of synovitis is well documented in terms of its histological features (4), its protein profile is not well comprehended.

Monocytes/macrophages play an important role in the pathogenesis of OA. Infiltration of monocytes into the joints onsets inflammation and propagation of the synovium and joint devastation in both the acute and the chronic phases of OA. Activated monocytes can mediate its infiltration into the affected synovium through its specific β2-integrins, including CD11c/CD18 and CD11b/CD18, binding with endothelial surface receptor [intercellular adhesion molecule-1 (ICAM-1) and vascular cell adhesion molecule-1 (VCAM-1)] (5). The chemotaxis of monocytes is directed through a concentration gradient of different chemokines, predominately, (C-C motif) ligand 2 (CCL2), which binds with its receptor, CCR2 (6). However, the infiltration of specific monocyte subtype and its functional commitment in OA synovium remain unclear.

In circulation, monocytes are classified into three main subtypes based on expression of cell surface receptors: CD14^++^CD16^–^ (classical: CM), CD14^++^CD16^+^ (intermediate: ITM), and CD14^+dim^CD16^++^ (non-classical: NCM) (7). Classical monocyte represents the major subtype of the monocyte population and is well known for its scavenging and inflammatory characteristics. This population is well equipped with a set of TLRs and scavenger receptors, capable of recognizing PAMPs, DAMPs, lipid, and dying cells *via* phagocytosis. Moreover, classical monocyte exhibits effector molecules, including various cytokines, myeloperoxidase, and superoxide, which facilitate the involvement of this population in various inflammatory processes (7). The intermediate monocyte subtype is poorly characterized and it shares many common chemokine receptors, such as CX3CR1 and CCR2, with classical and non-classical monocyte subtypes. This subtype demonstrates a pro-angiogenic behavior based on the expression of its surface markers including endoglin (ENG), TEK tyrosine kinase (Tie2, CD202b), and KDR (VEGFR2) (7). The monocyte heterogeneity has been further confirmed when CD16-positive monocyte differentiation is found within the CD14^+^ monocyte population and identified as a non-classical monocyte subtype. This monocyte population, which is known as patrolling monocyte phenotype, tends to interact with vascular endothelium through β2 integrin, lymphocytes function-associated antigen-1 (LFA-1), and CX3CR1. During tissue damage, the number of non-classical monocytes tends to be elevated up to 4-fold in circulation when compared with normal conditions (8).

In this study, the differential proteins in OA synovium as compared with proteins derived from synovium acquired from non-OA-diagnosed trauma patients are discovered. More importantly, the alteration in characteristics of the monocyte subtype in terms of its surface receptors and molecular expression, and phagocytic commitments in response to mediators derived from OA synovium is further delineated.

## Materials and methods

### Patients recruitment and sample acquisition

Approval was obtained from University of Malaya Medical Centre-Medical Research Ethics Committee (UMMC-MREC) (Ethics Number: 20164-2398) prior to conducting this study. The inclusion criteria for recruiting end-stage OA patients: individuals above 18 years old with primary OA only, who had been listed for a total knee replacement (TKR) at the UMMC. The inclusion criteria for recruiting non-OA-diagnosed trauma patients (trauma baseline control): individuals who had been listed for a lower limb trauma surgery (ACL reconstruction) at the UMMC and no evidence of OA based on arthroscopy evaluation. The exclusion criteria: individuals who had been listed for joint arthroplasty or arthroscopy, not within the UMMC; individuals who had a history of joint disease, such as lower limb OA, and listed for lower limb trauma surgery; individuals who had medical history of inflammatory diseases, such as asthma and autoimmune disorders; and individuals who had been taking anti-inflammatory drugs, especially non-steroidal anti-inflammatory drugs (NSAIDs). Informed consent was obtained before recruiting the patients for this study. Intra-operative samples of synovium from the same anatomical region were collected from patients undergoing primary arthroplasty surgery for end-stage knee OA (*n* = 8) and non-OA-diagnosed trauma patients (trauma baseline control—TBC) (*n* = 8) undergoing anterior cruciate ligament (ACL) reconstruction surgery. The demographic and clinical characteristics of patients are shown in Table 1. OA synovium conditioned media (OACM) was prepared following incubation of OA synovial tissue in the basic Dulbecco’s Modified Eagle Medium (DMEM; Gibco, United States) at a concentration of 500 mg tissue/mL for 72 h. OACM was harvested and centrifuged at 4,000*g* for 10 min to remove tissue residues. The supernatant was collected and stored at −80°C in aliquots of 1.5 mL for use in activation and migration analyses.

**TABLE 1:** Demographic and clinical characteristics of patients.

		OA	TBC
Sex	Male	3	6
	Female	5	2
Age		70.25 ± 9.28	26.00 ± 4.21
BMI		29.92 ± 3.15	N/A
CRP		8.57 ± 1.99	N/A
ESR		23.63 ± 1.88	N/A

N/A, Not available; BMI, Body Mass Index; CRP, C-reactive protein; ESR, Erythrocyte sedimentation rate.

### Proteomics and MALDI imaging

About 160 mg of pooled tissue samples either from OA or TBC, which were retrieved from −80°C, were treated with 8 mL of RIPA buffer combined with 1X Protease and Phosphatase inhibitor (GE Healthcare Life Sciences). The tissues were homogenized for 30 min and centrifuged for 5 min at 4°C to collect the supernatant. The pooled OA (pOAS) or pooled TBC (pTBCS) tissue supernatants were subjected to protein quantification using a BCA assay kit as per the manufacture protocol (BioVision, US). Prior to ESI-LCMS/MS QTOF acquisition, 1D SDS-PAGE was performed by loading various concentrations of pOAS and pTBCS and run parallel with BSA (reference) and standard ladder for 125 min. The gels were stained with a modified silver staining method as described by Yan et al. (9). Briefly, the gel was soaked in 2.5% silver nitrate containing soluble ions (Ag^+^) and subsequently developed by treatment with 6.25 g sodium carbonate and 100 μL of reductant. The process was halted with 3.65 g of EDTA, and finally, the gel was subjected to three washings using ddH_2_O water. The gel was imaged using UVP GelDoc. The identified protein bands of pOAS and pTBCS were cut into six sections before performing an in-gel tryptic digestion. All the gel sections were subjected to de-staining using 30 mM potassium ferricyanide before being treated with 0.4 μg of MS-grade trypsin solution and incubated overnight at 37°C. The supernatants derived from pOAS and pTBCS bands were collected after a brief centrifugation and acquired in an Agilent Accurate Mass Q-TOF 6550B Mass Spectrometer coupled with Agilent ChipCube using a standard protocol (10). PEAKS Studio (version 10, Canada) incorporated with the Swissprot database (Tax: *Homo sapiens*) was used for protein identification adopting an established protein analysis protocol (10). Decoy was constructed by the software to distribute the target-decoy database for PSM identification. The generated protein lists for every band for pOAS or pTBCS were consolidated into the respective groups and used the DAVID Functional Annotation Tools (Version 6.8, NIH, United States). A Venn diagram was prepared using the “Create-A-Venn” software. The STRING Functional Protein Association Network (Version 11, ©STRING Consortium 2019) was used to visualize the protein interaction networks. The gene ontology (GO) terms for Biological Process (BP) table generated from the STRING protein network were exported into an excel sheet and visualized using Reduce and Visualize Gene Ontology (REVIGO^1^). For MALDI imaging of OAS and TBCS, tissues were prepared according to a published method (11). Briefly, the serial sections from each frozen OAS and TBCS tissue sample were stained with hematoxylin and eosin (H&E) or thaw-mounted and fixed onto a MALDI plate. Photomicrographs of H&E-stained sections of OAS and TBCS were marked digitally. Histology-annotated optical images were merged to form a photomicrograph of the MALDI section. A crystalline matrix (20 mg/mL sinapinic acid in 1:1 acetonitrile/0.2% trifluoroacetic acid) was used based on a recommended concentration from a published method (11). A spray technique using an automated commercial system (ImagePrep, Bruker) was employed and matrix thickness, incubation time, and wetness were optimized. The tissue sections were acquired using the UltrafleXtreme (Bruker Daltonics, Billerica, MA) MALDI-TOF MS with an automated linear-mode acquisition method optimized for 2–20 kDa. SCiLS Lab (Version 2015b, Bruker Daltonic) was employed in the reconstruction of ion density images of the baseline corrected, normalized, and aligned MALDI MS spectral files. The identification of peptide signals was achieved by removing the chemical noise background (signal-to-noise ratio ∼ 3:1).

### Immunohistochemistry

Immunohistochemistry was performed to observe inducible nitric oxide synthase (iNOS) expression on OAS and TBCS tissues using an anti-iNOS primary antibody (GeneTex, Inc.) (1:500 dilution in Dako Antibody Diluent) Dako REAL™ EnVision™/HP, Rabbit/Mouse (ENV) secondary antibody. The staining was performed using the standard Dako REAL™ EnVision™ (Dako, Denmark) protocol from the manufacturer. The slides were scanned using a digital slide scanner (3DHISTECH, Hungary) and images were captured using CaseViewer 2.3 (3DHISTECH, Hungary).

### Transwell migration

Thirty milliliters of intravenous blood was drawn in trisodium citrate solution tubes from healthy donors (*n* = 6). The PBMNCs were isolated using the Ficoll-Pague density gradient (GE Healthcare Bio-Sciences, United States) and used in migration and flow cytometry analyses. About 1 mL of OACM was added in triplicate into a 12-well plate and the plate was inserted with a transwell. Monocyte Chemoattractant Protein-1 (MCP-1, 100 ng/mL) and serum-free media (SFM) were used as positive and negative controls, respectively. The PBMNCs were added in a transwell attached with a 3-μm pores diameter transparent insert membrane (IM) at the density of 3 × 10^6^/ml (Greiner Bio-One, United Kingdom). The plates were incubated for 3 h at 37°C in 5% CO_2_ with 95% humidity. The IMs were harvested and processed for scanning electron microscopy (SEM) imaging using an established protocol to view the migrated cells on the basolateral side (12). The migrated cells were captured using an inverted phase contrast microscope (Eclipse Ti-E, Nikon, United Kingdom) and the cells in the photomicrographs were calculated using the Image-J analysis software (IJ 151j/Java 1.8.2-64-bit, NIH, United States).

### Flow cytometry analysis

The pre- (PBMNCs) and post-migrated monocyte population were stained with two panels of eight-color conjugated monoclonal antibodies, Panel 1: anti-human CD45-BB515 (Clone HI30); anti-human CD3-PerCPCy5.5 (Clone UCHT-1); anti-human CD19-PerCPCy5.5 (Clone HIB19); anti-human CD56-PerCPCy5.5 (Clone B159); anti-human CD66b-PerCPCy5.5 (Clone G10F5); anti-human CD192-BV421 (CCR2, Clone 48607); anti-human CD182-PE (CXCR2, Clone 6C6); anti-human CD11b-BV510 (Clone ICRF44); Panel 2: All the antibodies as in the panel 1 were used in penal 2 except CD182/CXCR2 replaced with anti-human CX3CR1-PE (clone 2A9-1). The cell staining and flow cytometry acquisition in FACS Canto II (BD Biosciences, United States) were performed using an established protocol (13). The Median Fluorescence Intensity (MFI) was recorded and analyzed using the FlowJo software (BD, United States).

### PCR array and qPCR

Alterations in the relative gene expression of CSF1R, RANK (TNFRSF11A), MAPK1, S100A8, HSFB1, ITGAL, IL-1β, TNF-α, MYD88, NFATc1, IL13RA1, and CD93 were quantified either using a customize human RT^2^ Profiler PCR Array (catalog number: CLAH 25489) or conventional qPCR (Qiagen, United States). Results derived from the RT^2^ PCR array and conventional qPCR were evaluated using RT^2^ Profiler PCR Array Data Analysis version 3.5 and manual calculation. Expression data obtained were normalized to the average *C*_*T*_ value of the two housekeeping genes (HPRT1 and SDHA).

### Phagocytosis assay

The phagocytosis assay was performed using 1-μm Sulfate FluoSpheres^®^ latex beads (LB) (F8851, Invitrogen). The migrated monocytes in response to SFM, MCP-1, and OACM were collected and treated with LB at a ratio of 1:100 using a published protocol (14). The images captured using confocal laser microscopy (Leica TCS SP5 II, United Kingdom) were analyzed with the Leica Application Suite X imaging software (Leica LAS X, United Kingdom). For total LB fluorescence intensity detection, six random regions of interest (ROI) from three individual experiments were assigned for each interrogation and the corrected total cell fluorescence (CTCF) was calculated using the following equation (15):


(1) CTCF=Integrateddensity-(areaofselectedROI×fluorescenceofbackgroundreading)

where, integrated density and fluorescence of background reading were in arbitrary unit (A.U.) and area of selected ROI was in mm^2^.

### Statistical tools

The results acquired were averaged and presented as means ± standard deviation (SD). Statistical differences were determined by performing Student’s *t*-test or the Bonferroni correction test using the SPSS version 25 or GraphPad prism version 8 statistical tools. The differences were considered statistically significant if the value of *p* was < 0.05.

## Results

### Protein discovery

**FIGURE 1:**
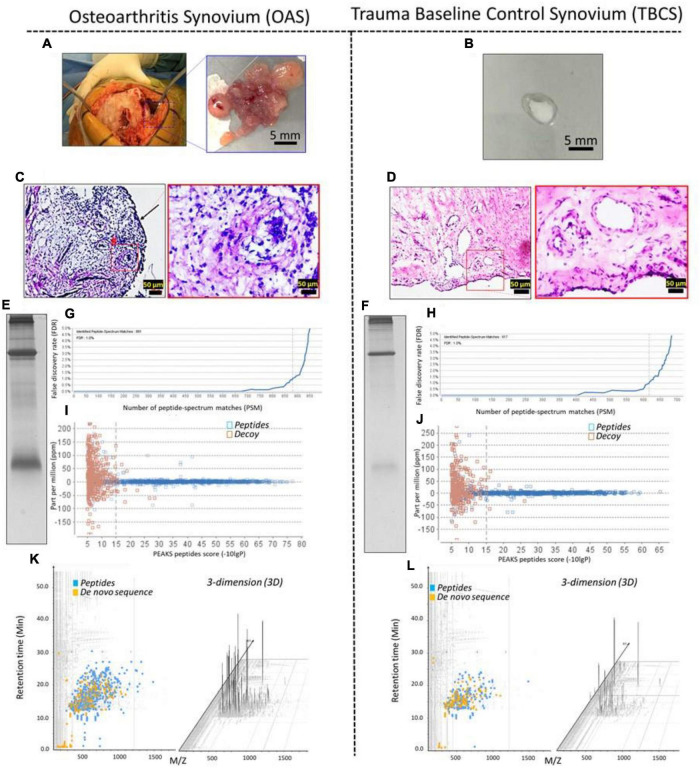
The profile of OA synovium (OAS) and trauma baseline control synovium (TBCS). **(A)** Synovium from the end-stage knee OA patient underwent knee arthroplasty. **(B)** Synovium from the sports trauma patient underwent arthroscopic surgery for anterior cruciate ligament (ACL) repair. **(C,D)** H&E stained microphotographs. **(E,F)** SDS-PAGE protein bands of pooled OAS or TBCS. **(G,H)** False discovery rate (FDR) curve of peptides matched with the corresponding ESI-LCMS/MS QTOF spectra of peptides. **(I,J)** Peptide spectrum matches (PSMs) score distribution of target (blue square—OAS or TBCS) and decoy (amber square). **(K,L)** LCMS distribution profile and 3D intensity peaks of peptide and *de novo* sequences.

The macroscopic evaluation indicates the presence of hypertrophy of synovial villi in OA synovium (OAS) (Figure 1A) but not in the TBC synovium (TBCS) (Figure 1B). The H&E histology section confirmed an increase in the thickness of the synovial lining layer (arrow) with a surge in the infiltration of mononuclear cells (#) and angiogenesis (Figure 1C) in OAS as compared to TBCS (Figure 1D). To discover the typical proteins present in the OAS as compared with TBCS, the synovial tissues from the same patient cohort were pooled to minimize inherent variabilities between patients and extracted proteins were separated using SDS-PAGE, as shown in Figures 1E,F (OAS) and (TBCS). The in-gel tryptic digested protein bands that were acquired using ESI-LCMS/MS QTOF for OAS and TBCS demonstrated mass spectra and its corresponding peptide matches with significant statistical score (*p* = 0.05/ −log_10_ 15) from the Swissport protein sequence database (Figures 1G,H). These peptide spectrum matches (PSMs) were further validated by comparing the PSMs of OAS and TBCS as indicated in blue squares with PSMs of decoy (amber squares). It was found that the PSMs of OAS and TBCS were scattered around the mass error of 0 and beyond the threshold level (dotted line, *p* < 0.05 = −log_10_ 15). It suggests a significantly higher peptide score with minimal mass error happened during peptides identification from the Swissport protein sequence database (Figures 1I,J). The peptides (blue square) and *de novo* (orange square) sequences identified from the protein database for OAS and TBCS were shown in an interactive LCMS distribution profile. The distribution of peptides and *de novo* sequences of OAS was distinct from that of TBCS (Figures 1K,L). The three-dimension (3D) peaks of identified peptides in OAS and TBCS confirmed the presence of peptides with different intensities (Figures 1K,L).

### Differential protein analysis

**FIGURE 2:**
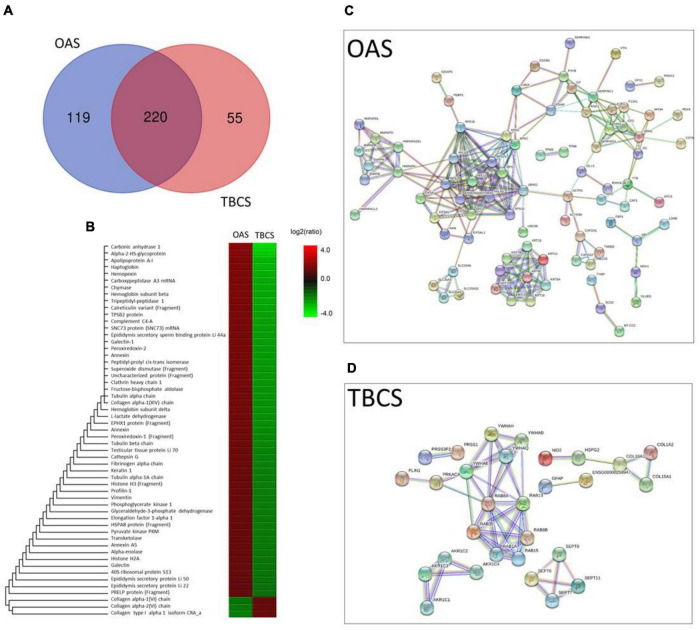
The differential protein analysis in OAS and TBCS. **(A)** Venn diagram; **(B)** Heatmap of overlapped proteins expressed at different magnitudes; **(C,D)** STRING protein–protein interaction networks.

The ESI-LCMS/MS QTOF displayed 339 and 275 proteins in OAS and TBCS, respectively (Supplementary Material 1). The Venn diagram shows about 119 typical proteins exclusively present in OAS, while 55 proteins in TBCS (Figure 2A). There were about 220 common proteins found in both synovium (Figure 2A). Among these overlapped proteins, 55 of them were found significantly higher in either group based on statistical analysis from the Peaks software. As shown in the heatmap, 52 proteins present in a greater magnitude in OAS as compared to TBCS, which showed only three proteins that were found in higher magnitude (Figure 2B). The STRING protein–protein interaction networks of differential protein profile demonstrate a unique protein network in OAS (Figure 2C) as compared to TBCS (Figure 2D). However, common protein–protein interaction networks were also found between these two cohorts (Supplementary Material 2).

### Functional protein analysis

**FIGURE 3:**
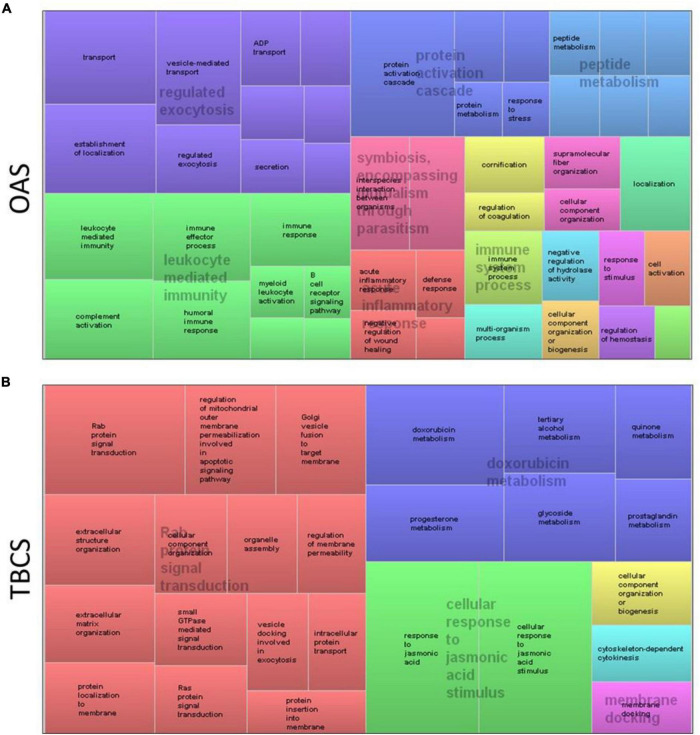
Treemap visualization of the enriched biological process obtained from REVIGO analysis of differential proteins. **(A)** OAS and **(B)** TBCS. The most enriched physiological processes corresponded with larger components on the map.

Using all 119 and 55 differential proteins found in OAS and TBCS, a treemap of enriched biological processes is illustrated in Figures 3A,B for OAS and TBCS, respectively. The most enriched biological processes are displayed as larger components/boxes. It was found that “regulated exocytosis” (Figure 3A, purple box) and “leukocyte mediated immunity” (Figure 3A, green box) were the predominant physiological processes found in OAS. In contrast, “Rab protein signal transduction” (Figure 3B, red box) occupied 50% of the treemap.

### Localized protein expression

**FIGURE 4:**
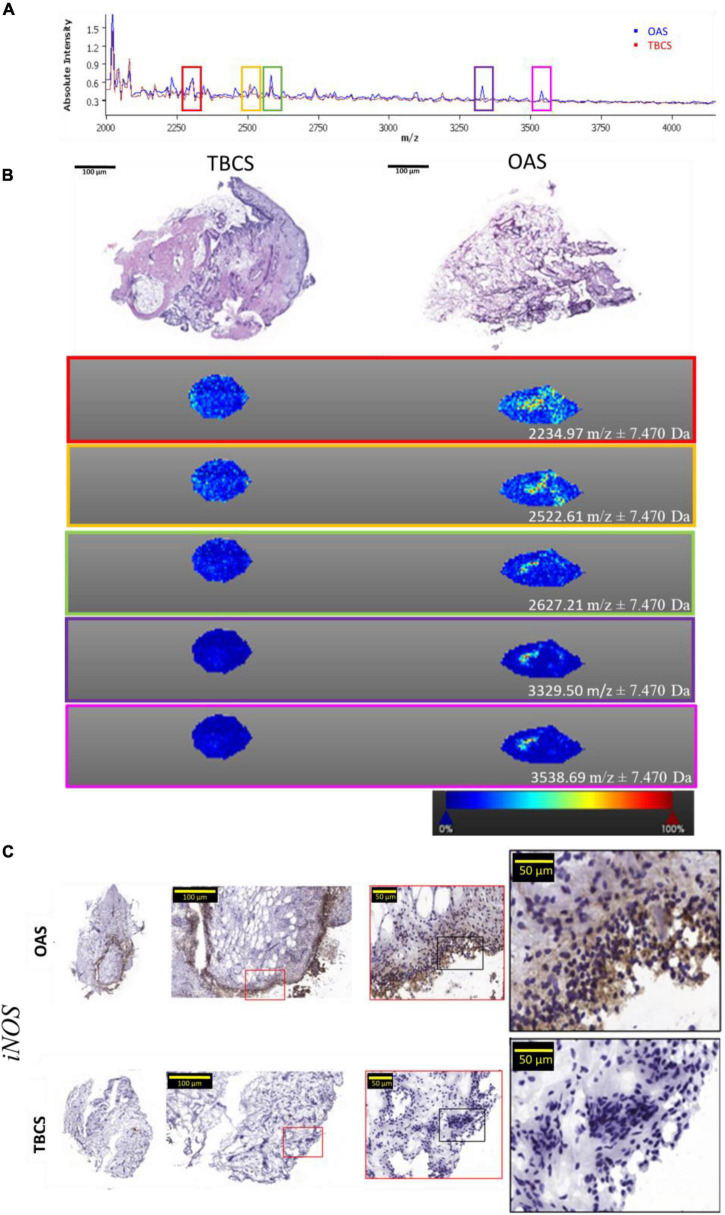
The localized protein profile in OAS and TBCS. **(A)** MALDI spectra; **(B)** H&E staining of histological section and its corresponding MALDI imaging; **(C)** Expression of the pro-inflammatory marker, *iNOS* in mononuclear cells.

The localized proteins present in OAS were discovered in comparison with proteins in TBCS. The proteins present on the surface were ionized using laser energy and they were separated based on their mass over ion ratio (*m/z*). The data were pre-processed in the SCiLS Lab by normalizing each spectrum according to its total ion count. Absolute intensities of the proteins found in OAS (spectrum in blue) and TBCS (spectrum in red) are shown in Figure 4A and Supplementary Material 3. About five co-localized proteins based on their outstanding peaks were further visualized using the SCiLS Lab MALDI imaging software. Figure 4B displays the localization of OAS and TBCS-specific protein masses of 2234.97 ± 7.470, 2522.61 ± 7.470, 2627.21 ± 7.470, 3329.50 ± 7.470, and 3539.69 ± 7.470 *m*/*z* that revealed a higher intensity in OAS as compared to TBCS. These localized proteins were predominately found in the hypertrophic area featured with an increased number of capillaries and migrated mononuclear cells as shown in the corresponding H&E histology section of OAS. These mononuclear cells were further explored for their pro-inflammatory profile based on iNOS expression. It was observed that the expression of the iNOS was aberrant in the intima region of OAS but absent in TBCS (Figure 4C).

### Migration potential analysis

**FIGURE 5:**
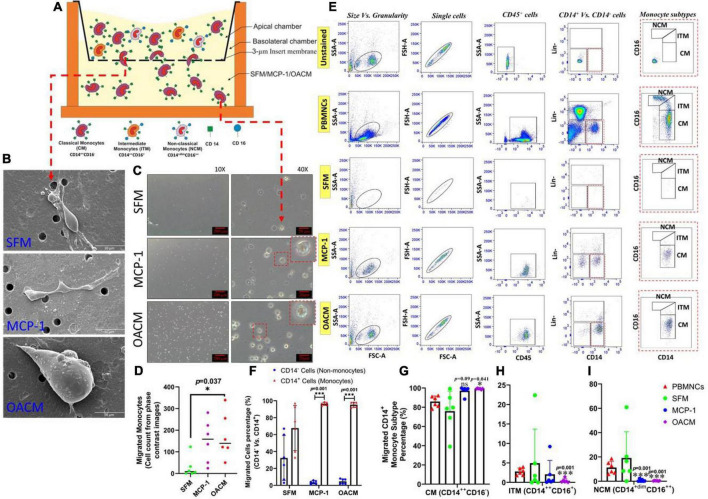
The migration potential of monocytes in response to SFM, MCP-1, and OACM. **(A)** Transwell migration model; **(B)** SEM micrographs of cells on the 3-μm pores insert membrane at basolateral site (post-migration); **(C)** Phase contrast photomicrographs of the migrated cells; **(D)** Enumeration of migrated cells from the phase contrast photomicrographs using Image-J; **(E)** The gating strategy in FACs analysis using CD45, Lineage negative (Lin-), CD14, and CD16 markers; **(F)** Percentage count of CD14^+^ (monocytes) and CD14^–^ (non-monocytes) gated migrated cell population; **(G)** Percentage count of migrated classical monocytes (CM: CD14^++^CD16^–^), **(H)** intermediate monocytes (ITM: CD14^++^CD16^+^)%; and **(I)** Non-classical monocytes (NCM: CD14^+dim^CD16^++^). PBMNCs were used as pre-migration baseline control when compared with migrated monocytes (not significant: ns). The assessments were reported to be statistically significant if *p < 0.05 and ***p < 0.001.

Monocyte migration potential in response to mediators derived from OAS was evaluated using a transwell migration model to mimic the pathophysiological condition in the synovium of OA patients (Figure 5A). The SEM micrographs show the mononuclear cells accomplished to migrate across the insert membrane *via* 3-μm pores from the apical chamber, in response to mediators, to the basolateral chamber (Figure 5B). The phase contrast photomicrographs confirmed an overwhelming migration in OACM and MCP-1 treated wells as compared with SFM (Figure 5C; 10 × magnification). The images captured at 40 × magnification indicate large eccentrically placed kidney bean shaped nucleated monocyte-like cells (Figure 5C). The number of cells enumerated from the phase-contrast photomicrographs indicated 6- and 5-fold increase in cell migration in response to OACM (*p* = 0.037) and MCP-1 (*p* = 0.096), respectively, when compared with SFM. The migrated cells were characterized using FACs for mononuclear cell markers. The side scatter-area (SSC-A) vs. forward scatter-area (FSC-A) showed a focal population of mononuclear cells and this population was comparable with the PBMNCs (Figure 5D). This mononuclear cell population in all groups was positive for CD45 hematopoietic lineage marker (Figure 5E). In addition, these CD45^+^ cells migrated in OACM, MCP-1 and SFM were found to be comprised of 95.30, 96.50, and 67.67% of CD14^+^ monocyte population, respectively, when compared with CD14^–^ non-monocyte population (Figure 5F). The monocyte subtypes either classical (CM:CD14^++^CD16^–^), intermediate (ITM: CD14^++^CD16^+^), or non-classical (NCM: CD14^+dim^CD16^++^) were identified using the classification consensus that has been approved by the Nomenclature Committee of the International Union of Immunological Societies (16). A significant migration of CM was observed with a proportion of the population more than 99% (*p* < 0.041) in response to OACM when compared with PBMNCs, which was 86% (Figure 5G). This OACM migrated monocytes were also comparable to monocytes migrated in response to single mediator positive control, MCP-1 with a total population of 97%. However, the migration of ITM or NCM in response to OACM was 0.4%, which was significantly lower (*p* = 0.001) when compared with PBMNCs, which comprised 3 and 11% of ITM and NCM, respectively (Figures 5H,I and Supplementary Material 4).

### Chemotaxis and inflammatory response

**FIGURE 6:**
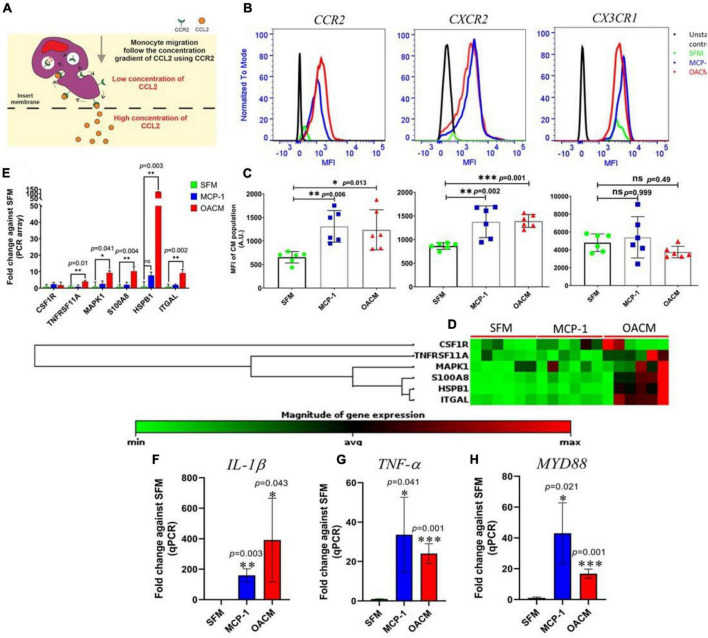
The chemotaxis and the pro-inflammatory response of monocytes to SFM, MCP-1, and OACM. **(A)** Transwell migration model of monocytes using CCR2 receptor binding with CCL2 in concentration gradient. **(B)** The flow cytometry histogram profile of CCR2, CXCR2, and CX3CR1 receptors. **(C)** The statistical analysis of the expression of CCR2, CXCR2, and CX3CR1. **(D)** Heatmap of gene expression array. **(E)** The fold-change in gene expression array. **(F–H)** Fold-change of gene expression in qPCR. The assessments were reported to be statistically significant if *p < 0.05, **p < 0.01 and ***p < 0.001.

Monocyte migration from peripheral into local tissue happens in chemotactic gradients orchestrated by the local tissue chemotactic cytokines/chemokines, such as CCL2 (MCP-1), CXCL8 (IL-8), or CX3CL1 (Fractalkine) (Figure 6A). The corresponding receptor binding with these cytokines/chemokines facilitates the rapid monocyte transendothelial migration. In this study, the expression of chemotactic receptors was measured only on the migrated CM monocyte population as the ITM and NCM population were below FACs sensitively level (Figure 6B). The CCR2 and CXCR2 expression on CM subtype in response to OACM were about 2.0-fold (*p* = 0.013) and 1.9-fold (0.011) significantly higher than that of SFM, respectively (Figure 6C). This finding was comparable with MCP-1 migrated CM subtype. However, the expression of CX3CR1was found to be moderate in both MCP-1 and OACM migrated CM subtype (Figure 6C and Supplementary Material 5). In PCR array analysis for genes associated with pro-inflammation, alarmins and cell adhesion in OACM migrated cells, the expression of TNFRSF11A (4.1-fold), MAPK1 (9.3-fold), S100A8 (10.3-fold), HSPB1 (121.5-fold), and ITGAL (9.2-fold) was found in higher magnitudes (Figure 6D and Supplementary Material 6) and statistically significant when compared with SFM migrated monocytes (Figure 6E). The qPCR analysis of pro-inflammatory and pathway genes, including IL-1β, TNF-α, and MYD88, in OACM migrated monocytes was 391. 5-, 24. 6-, and 16.8-fold significantly higher, respectively, when compared with SFM migrated cells (Figures 6F–H and Supplementary Material 7). When compared the gene expression of migrated monocytes in response to MCP-1 against SFM, 210. 6-, 33. 3-, and 63.6-fold significant increase in terms of IL-1β, TNF-α, and MYD88, respectively (Figures 6F–H) were observed.

### Phagocytosis functional analysis

**FIGURE 7:**
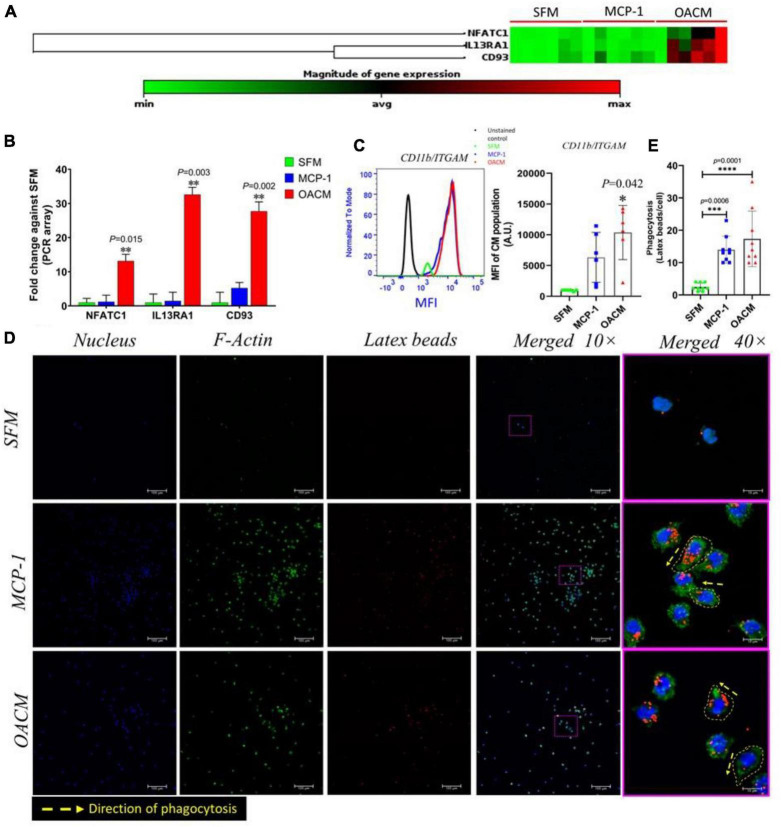
The phagocytosis potential of migrated monocytes in response to SFM, MCP-1, and OACM. **(A)** Heatmap of gene expression array of NFATC1, IL13RA1, and CD93 genes. **(B)** The fold-change of genes against monocytes migrated in SFM. **(C)** FACs analysis of CD11b/ITGAM receptor. **(D)** Confocal photomicrographs of monocytes internalized with latex beads at 10× and 40× magnification. **(E)** The quantitative analysis of latex beads uptake/cell by migrated cells in response to MCP-1 and OACM against SFM. The assessments were reported to be statistically significant if *p < 0.05, **p < 0.01, ***p < 0.001 and ****p < 0.0001.

The mechanistic study is important to understand the fate of the migrated immune cells from the peripheral into inflamed OAS. The heatmap of the gene expression array indicates a greater magnitude of expression of NFATC1, IL13RA1, and CD93, which are foreign body giant cell and phagocytosis-associated activity genes, were observed in monocytes migrated in response to OACM but not in MCP-1 or SFM (Figure 7A). The quantitative analysis of NFATC1, IL13RA1, and CD93 genes were (13.2-fold, *p* = 0.015), (32.6-fold, *p* = 0.003) and (27.7-fold, *p* = 0.002) significantly higher, respectively, when compared with SFM (Figure 7B). The surface adhesion molecule, integrin subunit alpha M (ITGAM)/CD11b FACs analysis shows a significant expression of this receptor on monocytes migrated in OACM when compared with SFM (*p* = 0.042). However, CD11b expression was marginal on monocytes migrated in MCP-1 (Figure 7C). The confocal photomicrographs of the phagocytosis assay confirm the fact that the migrated monocytes in OACM and MCP-1 enhanced the uptake of the latex beads (LB) (Figure 7D). The extension of the cellular filipodia to the direction of the LB for the phagocytosis was apparent at 40 × magnification in both OACM and MCP-1 groups (Figure 7D, indicated by a yellow dotted line and arrow). The quantitative analysis confirms that LB uptake/cell of migrated monocytes in OACM and MCP-1 was 6.9-fold *(p* = 0.0001) and 5.6-fold (*p* = 0.006) significantly higher than that of monocytes migrated in SFM (Figure 7E and Supplementary Material 8).

## Discussion

The histopathological analysis of the OA synovium confirmed that it was predominately enclosed by inflammation (17). Mounting evidence suggests that synovitis is the key aspect of the pathogenesis of OA (1, 18). However, the detailed characteristics of synovitis and its protein profile are not well comprehended. In this study, we adopted advanced proteomics and imaging tools to discover the protein profile in OA synovium. There is always a significant knowledge gap in the literature concerning the selection of an appropriate control group for OA synovium and considerable ethical challenges to acquiring such samples (19). However, there are a number of approaches available to fill this gap including using synovial samples acquired from cadaveric joints (20), above-knee amputation (21), arthroscopy for the unspecified indication (22), or even from normal-appearing areas of synovium within an OA joint (23). In this study, the non-OA-diagnosed synovium was obtained from sports injury patients, mostly young athletes who have had their anterior crucial ligament reconstruction 6 months before their injury was diagnosed. These patients were confirmed free from OA based on arthroscopic findings during ACL repair. The deprived and discarded synovium as part of the procedure during ACL repair were collected and used as non-OA-diagnosed synovium (trauma baseline control synovium). Although this approach is inferior to healthy synovium, however, there were several studies that reported that this approach can be used as acceptable baseline control (20, 24). The acquired OA synovium demonstrated a visible hypertrophic feature with thickening of synovial lining as a result of recruitment of mononuclear cells suggesting its local inflammatory condition. Accordingly, 119 differential proteins have been found in OA synovium as compared to the counterpart, the non-OA-diagnosed synovium, providing an avenue for further investigating the role of synovitis in OA progression.

The significantly upregulated proteins as portrayed in the heatmap confirmed the OA profile of synovium. This is because, the activities of carbonic anhydrase I and superoxide dismutase have been confirmed in OA progression (25, 26). However, to our knowledge, the remaining differential proteins found in OA synovium are yet to be fully understood in the context of OA onsets.

In this study, we found a typical protein network linking vitronectin (VTN), serpin family (SERPINC1 and SERPING1), and orosomucoid 1 (ORM1) in OA synovium. This was mainly because all these three proteins are often involved in the inflammatory vicious cycle enhancing the secretion of proinflammatory mediators, such as IL-1, TNF-α, and IL-6, by the immune cells (27, 28). Together with our findings and existing knowledge, inflammatory-driven synovitis has been confirmed. Therefore, a functional study is warranted, ideally using an animal model for OA progression where vitronectin, serpin family, and orosomucoid can be further investigated for their role in inflammatory pathway activation in synovium at different time points. This will provide an evidence to understand how these proteins coordinate the worsening of cartilage degeneration *via* upregulating the inflammatory pathways in OA.

We explored the Gene Ontology (GO) analysis of the biological process to gain a detailed biologic insight into differential proteins discovered in the OA synovium. It reveals leukocyte-mediated immunity (GO:0002443) as a main protein cluster in the OA synovium. This functional activity correlates with our H&E histology findings, where a significant infiltration of mononuclear cells was observed in the intima region of OA synovium but absent in the TBC synovium. This finding led us to investigate the localized proteins in OA synovium using a special imaging tool, matrix-assisted laser desorption ionization (MALDI). It reveals the images of unknown molecules, including 2234.97, 2522.61, 2627.21, 3329.50, and 3539.69 *m*/*z*. Although the related proteins to these spectra are yet to be fully understood, we found a spectrum with 3539.69 *m*/*z* was highly correlated with the alpha defensin protein family, which has the *m*/*z* between 3,300 and 3,500 (29). In OA synovium, this protein was found to be produced by B and NK cells. It tends to be involved in mRNA expression of TLR signaling molecules, transcription factor NF-κB, and pro-inflammatory enzyme, iNOS (30). This observation was supportive as we found the GO for B cell receptor signaling pathway (GO:0050853) and humoral immune response (GO: 0006959) in the OA synovium. Interestingly, the expression of iNOS exclusively present in the OA synovial lining further supported the correlation we made earlier. To further confirm the correlation between alpha defensin and iNOS expression in OA progression, a functional tissue-engineered synovium model can be adopted (31). This versatile biofidelic culture model can serve as a test bed to explore the biological role of alpha defensin in the OA synovium as compared with the healthy synovium. The shift in iNOS expression in response to increased concentration of alpha defensin, as well as pro-inflammatory mediators, can be studied in real time.

The second objective of our study was to investigate the alteration in characteristics of the monocyte subtype in terms of its surface receptors and molecular expression and phagocytic commitments in response to mediators derived from OA synovium. The transwell model used in this study was an excellent choice as it permits a reductionist approach that allows direct observation, quantitative analysis, and manipulation in ways that are currently impossible to attain in the living organism (32).

We observed from the SEM micrographs where the migration of monocytes across the 3-μm pores, which were three times smaller than an average size of monocyte, was notable. It allows us to infer the notion that the migration of monocytes was undertaken corresponding to the concentration gradient of chemotactic mediators in OA synovium, ruling out the effect of the gravitational pool. We attempted to explore the specific monocyte subtype, i.e., classical (CM), intermediate (ITM), or non-classical (NCM) that is responsible for supporting pro-inflammatory macrophage activity in OA synovium. It was evidenced that the migration of CM was significant in response to mediators in OACM. Interestingly, there was a study reported where depletion in CM numbers was significant in peripheral blood of patients with knee OA as compared with other ITM or NCM subtypes (33). This shortfall in CM number could be the reason for an extensive migration of CM in inflamed synovium as shown in our *in vitro* migration model. A similar pattern of CM depletion was also observed in another study when peripheral blood mononuclear cells (PBMCs) derived CM (82.5%) was compared with synovial fluid leukocytes (SFLs) (57%) in patients with knee OA. Interestingly, this study highlighted the notion that there was an increase in ITM subtypes from PBMCs (6.3%) to SFLs (39.4%). This 33.1% sharp hike in the ITM population could be contributed by 28% CM found to be depleted from PBMCs (34). This postulation was evidenced in another study where deuterium labeled ITM and NCM were found to emerge sequentially from the pool of CM (7, 35).

This was the first study that delineated the role of CM in OA synovium. In this study, the alteration in expression of surface receptors, such as CCR2 and CXCR2, in response to mediators from OACM was further investigated. The selection of these markers was made on the basis to understand the corresponding migration activity of monocytes. These markers are mainly involved in the chemotaxis activity of monocyte (36). The significant expression of these markers was parallel with the increased migration activity of CM in response to OACM. Our finding corroborates the previous report where monocyte recruitment was enhanced with increased expression of CCR2 (6). The significant expression of CXCR2 on CM also suggests the fact that CM may also be recruited *via* binding with IL-8 in OACM (37). However, whether these markers are the sole responsibility to drive this enhanced migration activity of CM needs to be further confirmed with an intervention study.

The stimulated monocytes can produce pro-inflammatory mediators including alarmins and cytokines to recruit excess monocytes from the circulation (33). Our finding evidenced that CM significantly upregulated its S100A8 and HSPB1 alarmins genes. This indicates the notion that CM might be undergoing stress conditions, thus partly responsible for the enhanced inflammation in the OA synovium. This finding was coherent with our GO for myeloid leukocyte activation found in the OA synovium-derived protein profile. Moreover, a similar outcome was also reported earlier where S100A8 increased the migration of pro-inflammatory Ly6C*^high^* monocytes, which is similar nomenclature for human CM phenotype, in knee OA synovium of a mouse model (38). This upregulation of inflammation can further enhance the production of pro-inflammatory cytokines, such as IL-1β (39) and TNF-α (40), and its downstream NF-κB pathway commitment *via* MYD88 adapter proteins. This notion was supported by our finding where a significant expression of MYD88 was found in CM migrated in response to mediators in OACM.

In this study, the gene expression profile also confirms the phagocytic functional commitments of CM in response to OACM. The expression of CD93/C1qR1, which is a cell adhesion surface protein responsible for the regulation of monocyte cell adhesion and phagocytosis (41), was significantly increased in CM migrated in OACM. This gene expression was parallel with the significant expression of surface integrin, CD11b/ITGAM subunit that could have facilitated the phagocytosis process of CM migrated in OACM as reported in a previous study (42). Interestingly, the enhanced uptake of latex beads by CM in the specific direction by extending its filipodia suggests the notion that the CM phagocytic role seemed to be pre-determined by the mediators present in the OACM. This finding was further supported by the GO for the defense response found in the protein profile of OA synovium. This finding highlights the fact that the role of phagocytosis is part of the defense activity of these professional phagocytic cells (43).

In a previous study, monocytes from patients with OA displayed increased osteoclastogenesis and bone resorption (44). This led us to explore the expression of osteoclast inducing gene, NFATC1 in monocytes migrated in OACM. Interestingly, we found that this population expressed a 13-fold higher NFATC1 gene as compared with the control group. This finding suggests the concept that the priming of osteoclast differentiation potential of monocytes in response to OA synovium-derived mediators is initiated. However, an osteoclast functional assay on the migrated monocyte population should be performed to further infer this notion.

## Conclusion

The differential proteins present in the OA synovium depict the pathophysiological condition of OA. This protein profile emphasizes the active role of immune cells in synovial tissue inflammation. This notion was clearly supported by the enhanced recruitment of monocytes, predominately classical subtype (CD14^++^CD16^–^). This population seems to be actively engaged in a phagocytic role to supplement the activity of local tissue macrophages. This pro-inflammatory phenotype could support the vicious cycle of synovial inflammation, thus wound healing process could be delayed and worsen the cartilage degeneration. Therefore, the CM subtype either can be used as a diagnostic tool for early detection of OA progression or the role of this population can be interfered to halt the onsets of OA in patients.

## Data availability statement

The datasets presented in this study can be found in online repositories. The names of the repository/repositories and accession number(s) can be found in the article/Supplementary material.

## Ethics statement

The studies involving human participants were reviewed and approved by the University of Malaya Medical Centre-Medical Research Ethics Committee (UMMC-MREC), Kuala Lumpur, Malaysia. The patients/participants provided their written informed consent to participate in this study.

## Author contributions

TK and KA supervised the study and acquired the funding. NS and KG conceptualized, analyzed data, investigated, and wrote the original draft. NS, KG, IO, SA, MR, and HR contributed to the methodology. CC, KA, MA-F, and ST recruited donors and acquired clinical samples. All authors have approved the final version of the manuscript.

## Footnotes

1
http://revigo.irb.hr
