# Supplementary material for: Typical response of CD14++CD16– monocyte to knee synovial derived mediators as a key target to overcome the onset and progression of osteoarthritis
Source: Front Med (Lausanne). 2022 Aug 29;9:904721. doi: 10.3389/fmed.2022.904721 (PMC9464827; doi:10.3389/fmed.2022.904721)
Supplement: Supplementary file 2 [file Data_Sheet_2.DOCX]

**Extended Methods**

1. Proteomics using ESI-LCMS/MS QTOF
   1. Protein extraction

Protein lysates from each groups were pooled to minimize inherent variability across tissue samples derived from different donors, and to obtain a consistent protein profile. The tissues were washed briefly with chilled 1X phosphate buffered saline (PBS) to remove any blood, whilst keeping it on ice. Each eight tissue samples from both groups (OA and TBC) were cut into relatively equal volumes (approximately 20 mg each) and samples were pooled into their respective tubes, labelled pooled OA and pooled TBC. In general, 500 µL of RIPA buffer with 1X Protease and Phosphatase inhibitor (GE Healthcare Life Sciences) was added for approximately every 10 mg of tissue. Since we have 160 mg of tissue in total, 8 mL of RIPA buffer with inhibitor was added to each pooled tubes. The pooled tissues containing buffer and inhibitor were then transferred to a tissue homogenizer to thoroughly homogenize the samples while keeping the process on ice for 30 min. The homogenization was carried out until the solution was murky and the tubes containing homogenized solution of both pooled OA and pooled trauma tissues were then centrifuged at 500 g for 5 min in 4°C. Then, the supernatant containing the processed protein lysate was collected and stored in -20°C for short term storage until further use in the subsequent parameter.

- 1. Protein quantification

Protein quantitation was conducted using BCA Assay Kit from BioVision Incorporation according to the manufacturer’s instruction. Working solution was prepared by making a 1:50 of copper reagent and BCA reagent. For the preparation of BSA standard curve, eight tubes were labelled. The BSA standard was diluted to 1 mg/mL stock solution. The same buffer contained in samples was used to prepare BSA solutions which was RIPA buffer. The serial dilutions were prepared. Few concentrations of the protein lysate from both pooled OA and TBC were diluted to 1:150, 1:100, 1:50, 1:25 and 1:10 so that they fall within kit detection range which is 0.01 – 0.06 mg/mL. A 50 µL of standards and samples were pipetted into 96-well microtiter plate in technical triplicates followed by the addition of 100 µL of working solution into each well containing the standard and samples. The plate was gently shaken to mix and incubated for 1 h at 37°C. The optical density (OD) was measured at 562 nm. After obtaining the OD, a BSA standard curve was generated based on the absorbance reading against the BSA concentrations, which later was used to determine the protein concentration in both of the samples by subtracting the blank value of Tube 1 as it contains buffer used for their preparations.

- 1. Protein separation using 1D SDS-PAGE

In order to check for the presence of proteins prior to ESI-LCMS/MS QTOF acquisition, SDS PAGE was carried out to separate the proteins based on their molecular weights. A 12.5% SDS PAGE was performed according to the standard protocol. Samples from both pooled OA and pooled TBC with different concentrations were prepared by mixing with equal volume of sample buffer which later were boiled at 80°C for 5 min. A 5 µg/mL BSA was used as a reference. Before casting the gel, leakage test was conducted by pouring ddH_2_O into the space between the plates and let to stand for 5 min. If there was no leak detected, freshly prepared resolving gel solution was poured slowly into the casting frame and was let to polymerise for 1 h. After 1 h, the top of the resolving gel was dried with filter paper before pouring the stacking gel solution on top of resolving gel till it overflowed. The comb was immediately inserted and was let to polymerise for 1 h. After the polymerization, the gels were transferred to the electrophoresis tank and were assembled according to the manual. The mini tank was filled with running buffer before loading the samples. A 10 µL of ladder, 20 µL of 5µg/mL BSA and 20 µL of the prepared samples of varying concentrations were loaded into each well using gel loading tips. The electrophoresis started with 50 V for the first 20 min and was increased to 100 V for approximately 1 h 45 min until the indicator dye reach the end of gel. The gels were stained with modified silver staining method. The silver-stained images were scanned and saved using UVP GelDoc for reference.

- 1. In-gel tryptic digestion

The 1D SDS-PAGE gel was rinsed with ddH_2_O and six bands containing all of the proteins separated based on their molecular weight, were cut from the gel using a cleaned scalpel. Since the proteins had been separated into different bands according to their molecular weights, six bands were collected from each sample based on the gel shown in Figure 1. The gel pieces were placed in the siliconized microcentrifuge tubes (Eppendorf, Germany) and labelled accordingly to reduce the binding effect of peptides of the tube surface. Prior to trypsin digestion, additional de-staining step is required for silver staining. De-staining solution was prepared by mixing 30 mM potassium ferricyanide and 100 mM sodium thiosulfate in a 1:1 ratio just before use. The gel was immersed in the de-staining solution until the brownish colour disappear within 3-5 min. Then, the gel was washed with ddH_2_O thee times for 10 min each or until the yellow colour 29 was removed. After that, the gel pieces were covered with 200 µL of 200 mM ammonium bicarbonate (ABC) in 40% acetonitrile (ACN) and incubated at 37°C for 30 min. The supernatant from each tube was discarded. A 200 µL of reduction buffer (DTT) was added to each tube containing gel pieces and incubated for 1 h at 56°C. Then, all of the tubes containing the gel pieces were centrifuged briefly for 10 secs and all solution were discarded. A 200 µL of alkylation buffer (IAM) was added to the gel pieces and incubated for 30 min in the dark at RT. The liquid was then discarded from the tubes. After reduction and alkylation, the gel pieces were washed with 200 µL of 50 mM ABC for 15 min at RT and the liquid discarded. The previous step was repeated with 50 mM ABC in 50% ACN. A 200 µL of pure ACN was used for washing the gel pieces a third time for 15 min at 37°C before centrifuging the gel pieces for 10 secs followed by discarding the liquid. A 20 µL containing 0.4 µg of MS grade trypsin solution was added to the gel pieces for trypsin digestion and incubated at RT for 5 min. Afterwards, 200 µL of 40 mM ABC in 9% ACN was added to each gel pieces ensuring that all of them were covered with the solution. The gel pieces containing trypsin were incubated overnight at 37°C after which the tubes were briefly centrifuged for 10 secs. Afterwards, supernatant from each tubes were collected to their new tubes that were labelled as “Collection Tube” (CT). A 200 µL of 5% FA was added to the gel pieces, vortexed briefly and incubated at 37°C for 15 min before recovering the supernatant to their respective CT. The previous step of adding, vortexing, incubating and recovering supernatant were repeated twice for 200 µL of 50% ACN in 5% FA and 200 µL of pure ACN, accordingly. After extracting the peptides and discarding all of the gel pieces, the collection tubes were left in the vacuum concentrator for overnight to completely dry the 30 recovered peptides. The lyophilized samples were collected the next day and stored in - 20°C before analyse using MS.


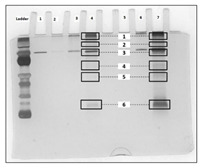


Figure 1: Selection of regions/bands of interest from SDS PAGE gel.

1. Proteomics using MALDI MSI
   1. Sample processing

A 10 µm thickness serial sections were cut from each frozen tissue sample and placed directly onto conductive indium-tin oxide (ITO) glass slide, which can accommodate multiple tissue sections on the same slide. However, the centre area of the slide (approximately 1 cm wide) was kept empty for proper functioning of the optical sensor of the Image Prep. ITO slide carrying the tissue sections was washed in ddH_2_O for 1 min followed by washing with ddH_2_O and fixing in graded ethanol of 70% and 100% for 1 min each. Then, the slide was allowed to dry in a vacuum desiccator for at least 15 min. Polysine adhesion slides (Thermo Fisher Scientific, England) was used to mount the adjacent section for H&E staining following standard protocols of graded ethanol and xylene dehydration and was stored in a dry atmosphere before viewing under light microscope.

- 1. Automated matrix spraying

In this present study, the deposition of matrix solution on ITO slide carrying tissue sections was accomplished using an automated matrix sprayer, that is, ImagePrep (Bruker Daltonics, Germany). A 25 mg/ml sinapinic acid in 1:1 acetonitrile:0.2% TFA was prepared. To start the matrix deposition on the sample, the following steps were carried out. First, the 10 mL bottle was filled with approximately 5 mL of matrix solution and remounted into the ImagePrep instrument. Then, the ITO slide was placed on the elevated rectangular area on the bottom of the ImagePrep spray chamber so that the sensor window was not covered by sample material. In the ImagePrep station, matrix application was fully automated with 30-100 cycles within 1 h, with an average droplet size of ~20 µm was deposited in layer, incubated in saturated atmosphere and was allowed to dry.

- 1. MALDI MSI acquisition

UltrafleXtreme (Bruker Daltonics, Billerica, MA) MALDI-TOF MS was used in this study to acquire profile spectra. It was set to run using the default setting of automated linear-mode acquisition method optimized for 2-20 kDa. BSA was used as reference for calibration purposes. Data acquisition was first optimized for the detection of calibrator peaks and no further adjustments were made during analysis. SCiLS Lab (Version 2015b, Bruker Daltonic) was employed in the reconstruction of ion density images of the baseline corrected, normalized, and aligned MALDI MS spectral files.

- 1. Immunohistochemistry (IHC) staining of iNOS

To further validate the inflammatory condition in OA synovial membrane, IHC was carried out using Dako REALTM EnVisionTM Detection System, Peroxidase/DAB+, Rabbit/Mouse (Dako Denmark) by looking at the iNOS expression. The frozen tissue sections of 10 µm thickness were placed onto polysine slides (Thermo Scientific^TM^) and stored in a sealed slide box at -80°C until further use. Prior to immunostaining, the tissue 35 sections were immersed in the fixative solution of pre-cooled acetone for 10 min. Then, the acetone was drained off and allowed to evaporate from the tissue sections at RT for approximately 20 min. After rinsing off the acetone with 1X PBS, sections were incubated in hydrogen peroxide solution (Abcam, USA) at RT for 10 min before rinsing with the PBS again, twice for 5 min each. In order to permeabilize the membrane, 100 µL blocking buffer containing 1% Tween 20 was added onto the sections and incubated in a humidified chamber at RT for 1 h. Prior to adding the antibody, a circle was drawn around the tissue section using a hydrophobic pen (Dako, Agilent). A 100 µL of diluted anti-iNOS antibody (GeneTex, Inc.) (1:500 dilution in Dako Antibody Diluent) was added to the sections enclosed by the circle made using the Dako pen, and incubated in a dark humidified chamber overnight at 4°C before rinsing off the excess antibody with 1X PBS for 2 changes, 5 min each. Then, few drops of Dako REAL™ EnVision™/HP, Rabbit/Mouse (ENV) were added and incubated in the same chamber at RT for 30 min in the dark. After rinsing off the secondary conjugated antibody with PBS twice for 5 min each, 100 µL of substrate working solution (CHOM) was added to the sections on the slides for approximately 10 min until a brownish color appeared. Before counterstaining the sections with hematoxylin, the slides carrying the tissue sections were washed once again with PBS. Counterstaining was done by immersing slides in hematoxylin for 90 secs followed by gentle rinsing for 15 min with running tap water. The slides were then dehydrated though degraded concentrations of alcohol (95%, 95%, 100% and 100%) for 5 min each before clearing in xylene twice for 1 min each and mounted with coverslip before observing under microscopy.
